# Mucormycosis and Hearing: A Hospital-Based Study

**DOI:** 10.1055/s-0045-1802578

**Published:** 2025-08-07

**Authors:** Monalisa Jati, Ripu Daman Arora, Ruuzeno Koutsu, Nitin M. Nagarkar, Roshan Marandi, Kartik Agrawal

**Affiliations:** 1 Department of Ear, Nose, and Throat – Head and Neck Surgery, All India Institute of Medical Sciences, Raipur, Raipur, Chhattisgarh, India; 2 Department of Ear, Nose, and Throat – Head and Neck Surgery, All India Institute of Medical Sciences, Guwahati, Guwahati, Assam, India; 3 SRM Medical College Hospital and Research Centre, SRM Nagar, Potheri, Chengalpattu, Tamil Nadu, India; 4 School of Public Health, All India Institute of Medical Sciences, Raipur, Raipur, Chhattisgarh, India

**Keywords:** mucormycosis, hearing loss, auditory system, middle ear dysfunction, pure-tone audiometry, acoustic immittance

## Abstract

**Introduction** Mucormycosis, a fungal infection with severe consequences, gained prominence during the coronavirus disease 2019 (COVID-19) pandemic. While significant efforts were made to understand the systemic implications of mucormycosis, its effect on the auditory system remains unexplored.

**Objective** This study aimed to investigate the auditory implications of mucormycosis through a comprehensive case-control study focusing on hearing loss and middle ear dysfunction.

**Methods** A total of 30 mucormycosis patients without prior auditory issues, and 30 age and gender-matched controls, underwent comprehensive hearing assessment, including pure-tone and immittance audiometry. Statistical analyses, including descriptive statistics, Mann-Whitney U test, Pearson chi-square test, and multiple logistic regression were performed to analyze the data.

**Result** Mucormycosis patients exhibited significantly higher auditory thresholds across all frequencies compared to controls ( *p* < 0.05). Approximately half of these patients experienced some degree of hearing loss, predominantly mild. Immittance measurement showed a higher prevalence of abnormal tympanograms in mucormycosis patients, indicating middle ear dysfunction. Acoustic reflex was absent in half of the mucormycosis patients suggesting impaired auditory function.

**Conclusion** The present study revealed significant auditory health impacts of mucormycosis, finding a notable prevalence of hearing loss and middle ear dysfunction, which emphasizes the need for routine audiological evaluations and increased awareness of mucormycosis-related auditory issues. Despite the study's limitations, we identified potential risk factors for hearing impairment, suggesting a need for further large-scale studies to confirm these findings and understand the mechanisms. These insights aim to improve diagnosis, treatment, and prevention strategies for better clinical outcomes.

## Introduction

Mucormycosis, commonly known as zygomycosis, garnered substantial attention during the coronavirus disease 2019 (COVID-19) pandemic due to its rapid spread and potentially life-threatening nature. This fungal infection is known for its extensive angioinvasion, rapid host tissue destruction, and swift dissemination. It often affects individuals with predisposing conditions, such as uncontrolled diabetes, immunosuppression, iron overload, and organ and hematopoietic transplantation. 1 2 3 4 The most common clinical manifestation is rhino-orbital-cerebral mucormycosis, impacting the sinuses, orbit, cranial cavity, and associated structures within the head and neck region. 5 6

Globally, mucormycosis incidence varies from 0.005 to 1.7 cases per 1 million individuals, while India stands out with a prevalence of 0.14 per 1 thousand cases, which is 80-fold higher than that in developed nations. 7 8 9 Amidst this unsettling scenario, the infection rapidly escalated, with a mortality rate surpassing 50%, emphasizing the urgent need for a comprehensive understanding of mucormycosis and its diverse manifestations. 4 10

Despite extensive research, mucormycosis still lacks adequate exploration of its impact on the auditory system and resultant hearing loss, which can significantly impair the quality of life. 6 11 12 Although rare, this infection can severely affect the auditory system, particularly in immunocompromised individuals, with documented case reports of temporal bone involvement. 13 14 15 16 These cases presented with symptoms such as otitis externa, ear pain, purulent otorrhea, and hearing loss leading to middle ear polyps and granulation tissue. 14 15 16 Some middle ear cases also presented with facial palsy and otocerebral involvement. 16 In nondiabetic patients, non-invasive mucormycosis can cause conductive hearing loss and, in severe cases, cholesteatoma due to angioinvasion, vessel thrombosis, and tissue necrosis. 17 Studies also suggested a potential risk of fungal spores infiltrating middle ear structures via the Eustachian tube, resulting in conductive hearing loss. 18 While rare, ear involvement in mucormycosis can be severe, underscoring the necessity of addressing auditory issues post-primary treatment for mucormycosis.

Our current understanding of the auditory consequences of mucormycosis is limited, based on individual case reports and small case series studies. 14 17 18 19 Addressing this knowledge gap requires urgent, extensive studies and clinical trials with rigorous methodologies to establish a solid foundation. Therefore, the present study aims to explore these auditory implications through a case-control study, enhancing understanding of early detection and intervention, particularly crucial in regions experiencing a surge in mucormycosis cases, like India. This endeavor will bolster our comprehension of the infection's impact on the auditory system and assist healthcare professionals in devising enhanced approaches for diagnosis, treatment, and prevention.

## Methods

This case-control study was conducted at a tertiary care center in Raipur to investigate auditory implications associated with mucormycosis. Ethical approval (AIIMSRPR/IEC/2021/1015) was obtained from the institute's Ethics Committee before commencing the study.

Initially, a total of 50 patients clinically diagnosed with mucormycosis were considered for this study. However, 20 individuals with a history of hearing loss before the onset of this infection were excluded. The remaining 30 patients, who did not report any previous auditory disease, hereditary hearing loss, ototoxic drug intake, head trauma, chronic noise exposure, neurological conditions, autoimmune disorders, vascular diseases, or metabolic disorders, were carefully selected for the test group. In this cohort, mucormycosis was confirmed by a combination of tissue biopsy and computed tomography (CT) scans of the lungs and sinuses. Among these 30 patients, 22 (comprising 16 males and 6 females) developed mucormycosis after the COVID-19 infection, while the remaining 8 (consisting of 5 males and 3 females) were independent cases. This test group was specifically chosen from the age range of 20 to 49-years-old, with a mean age of 40.9 ± 6.7, to minimize potential age-related effects on the auditory system.

A control group of 30 age (mean age of 40. ± 7.3 years) and gender-matched healthy individuals was formed for a comparative analysis. These participants were selected using a combination of convenience and random sampling methods. These individuals were enrolled if they met the inclusion criteria of not having a clinical diagnosis of mucormycosis and did not exhibit any of the exclusion criteria outlined earlier.

All the participants willingly consented to the study. Before initiating treatment, a hearing assessment was conducted to explore the link between mucormycosis and auditory health in this group.

### Hearing Assessment

A comprehensive hearing assessment was performed, which encompassed a detailed case history, examination of the ear, nose, and throat (ENT) region, and a thorough audiological evaluation. Before the hearing evaluation, an otoscopic examination ruled out outer ear issues. The audiological evaluation was conducted in a soundproof room using pure-tone audiometry (PTA) and acoustic immittance. This dual approach of audiological evaluation provided nuanced insights into auditory pathology, enhancing sensitivity in detecting and analyzing mucormycosis-related hearing impairment.

#### Pure-Tone Audiometry

Pure-tone audiometry, the gold standard for assessing hearing thresholds, determined hearing thresholds across various frequencies, aiding understanding the degree and type of hearing loss. 20 The MA-42 (MAICO Diagnostics GmbH, Berlin, Germany) diagnostic audiometer with a Sennheiser HDA 300 (Sennheiser Electronic GmbH & Co. KG, Wedemark, Germany) headphone was used to assess air conduction thresholds from 250 Hz to 8 KHz, employing the modified Hughson-Westlake method. 21 Bone conduction thresholds from 250 Hz to 4 KHz were measured using a Radioear B71 (RadioEar, Middelfart, Denmark) bone vibrator.

#### Acoustic Immittance Measurement

Immittance audiometry assesses middle ear function, identifying conductive hearing loss and middle ear abnormalities. 22 These measurements were conducted using a middle ear analyzer (Interacoustics, model AT235, Middelfart, Denmark), with a 226-Hz probe tone with pressure levels ranging from +400 daPa to -600 daPa, with a constant intensity of 85 dB SPL.

The results were recorded and inputted into a Microsoft Excel (Microsoft Corp., Redmond, WA, USA) spreadsheet and the IBM SPSS Statistics for Windows (IBM Corp., Armonk, NY, USA) software, version 27.0, was used for a comprehensive analysis.

### Statistical Analysis

The data were analyzed using the IBM SPSS Statistics for Windows, version 27.0. A descriptive analysis was employed, with categorical variables expressed as percentages and continuous variables as medians and interquartile ranges. Normality was assessed using the Shapiro-Wilk test. Due to the non-normal distribution, bivariate relationships were analyzed using the Mann-Whitney U-test. Categorical variables were analyzed using the Pearson chi-square test with a 95% confidence interval (95%CI). Multiple logistic regression models were employed to control for potential confounders and enhance statistical validity. A significance level of *p* < 0.05 was chosen to determine the statistical significance.

## Results

### Audiometric Analysis: Controls versus Mucormycosis

**Fig. 1:**
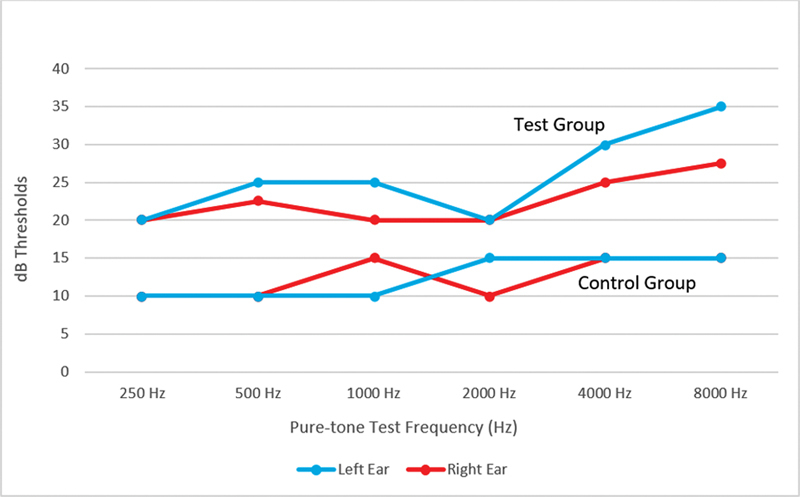
Frequency graph of the pure-tone audiometric threshold medians for the control and test groups.

The study compared hearing thresholds across different frequencies in the mucormycosis and control groups. Results consistently showed higher median thresholds in the mucormycosis group across all frequencies for both ears, with wider interquartile ranges (IQRs; first to third quartiles, Q1–Q3) at higher frequencies (4 and 8 KHz), indicating greater variability ( Table 1 ). The statistical analysis confirmed significant differences ( *p* < 0.05) between groups. Fig. 1 visually illustrates these findings, emphasizing mucormycosis's detrimental effect on hearing, especially at higher frequencies. These findings underscore the importance of regular audiometric assessments for managing mucormycosis-related hearing loss.

**Table 1:** Descriptive analysis of audiometric thresholds: control group versus test group (mucormycosis patients)

Ear/auditory frequency (Hz)	Auditory threshold in dB (quartiles, Q)						
	Control group			Test group			
	Median (Q2)	Q1	Q3	Median (Q2)	Q1	Q3	*p* -value
R 250 L 250 R 500 L 500 R 1000 L 1000 R 2000 L 2000 R 4000 L 4000 R 8000 L 8000	10 10 10 10 15 15 10 15 15 15 15 15	10 10 10 10 10 10 10 10 15 13.75 10 10	11.25 10 15 15 15 15 15 15 16.25 16.25 15 20	20 20 22.50 25 20 25 20 20 25 30 27.50 35	15 20 20 20 15 20 15 15 20 20 20 25	25 26.25 25 31.25 26.25 35 25 30 36.25 36.25 40 43.75	< 0.001 < 0.001 < 0.001 < 0.001 < 0.001 < 0.001 < 0.001 < 0.001 < 0.001 < 0.001 < 0.001 < 0.001

**Abbreviations:** L, left; R, right.

**Notes:** Left and right ear median values (Q2), Q1 (lower quartile), and Q3 (upper quartile) were calculated for each participant for analysis. Mann-Whitney U-test was used to compare the sub-groups and statistically significant p values were obtained at all the auditory frequencies.

### Status of Hearing Loss among Mucormycosis Patients

Analysis of hearing loss distribution among mucormycosis patients revealed that nearly half of the 30 patients ( *n* = 14, 46.67%) had no hearing loss. Among those with hearing loss ( *n* = 16, 53.33%), the majority were mild ( *n* = 12), with 7 having unilateral and 5 bilateral losses. Furthermore, two patients each had moderate (one unilateral and one bilateral) and moderately severe (one unilateral and one bilateral) hearing loss ( Table 2 ).

**Table 2:** Degree and laterality of hearing loss among mucormycosis patients ( *n* = 16)

Degree of hearing loss	n	Unilateral	Bilateral
Mild	12	7	5
Moderate	2	1	1
Moderately severe	2	1	1
Total	16	9	7

### Determinants of Hearing Loss in Mucormycosis Patients

After meeting the primary objective, an extensive analysis was conducted to discern the determinants of hearing loss in the study population. Both chi-square test and multiple logistic regression analysis were employed, considering various factors such as affected side, age, gender, COVID-19 status, and mucormycosis type as the determinants ( Table 3 ). However, neither analysis revealed a statistically significant association between hearing loss and the examined variables. While certain variables such as age (< 35 years), mucormycosis side (left), and type (rhino-orbital) displayed slightly elevated adjusted odds ratios in the regression analysis, none reached statistical significance. These findings imply potential associations warranting further research with larger sample sizes to elucidate underlying mechanisms.

**Table 3:** Determinants of hearing status in mucormycosis patients through multiple logistic regression analysis

Variables		Hearing Status		N of ears ( *n* = 60)	OR (95% CI)	*p* -value	AOR (95% CI)	*p* -value
		Normal	Hearing loss					
**Side of mucormycosis**	**Left**	23	11	34	2.091 (0.730–5.989)	0.167	3.204 (0.909–11.297)	0.070
	Right*	13	13	26				
**Age**	**< 35**	7	29	36	1.690 (0.391–7.308)	0.480	1.595 (0.281–9.048)	0.598
	≥ 35*	3	21	24				
**COVID-19**	**Present**	27	17	44	1.235 (0.388–3.938)	0.721	1.684 (0.445–6.381)	0.443
	Absent*	9	7	16				
**Gender**	**Male**	24	18	42	1.500 (0.473–4.761)	0.490	1.653 (0.446–6.132)	0.452
	Female*	12	6	18				
**Type of mucormycosis**	**Rhino-orbital**	16	16	32	0.400 (0.137–1.170)	0.091	0.228 (0.043–1.192)	0.080
	Sino nasal*	20	8	28				

**Abbreviation:** AOR, adjusted odds ratio; CI, confidence interval; COVID-19, coronavirus disease 2019; OR, odds ratio.

**Note:** *Reference category.

### Immittance Analysis: Control versus Mucormycosis

**Fig. 2:**
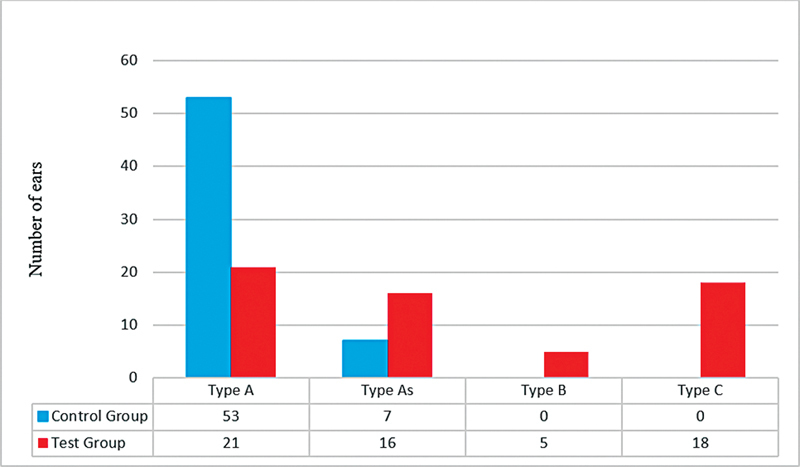
Immittance findings for both ears: control group versus test group (mucormycosis patients).

Combined pure-tone audiometry and immittance measurements confirmed middle ear dysfunction. Immittance audiometry delineated admittance and peak pressure, classifying various tympanogram types. The control group (60 ears) showed abnormal tympanograms in 7 ears, contrasting sharply with the test group's (60 ears) higher prevalence of abnormal tympanograms, with 39 ears. In the test group, A-type tympanograms were present in 21 ears, As-type in 16, B-type in 5, and C-type in 18 ears. Conversely, the control group primarily exhibited A-type (53 ears) and As-type (7 ears) tympanograms, with none demonstrating B or C-type, as depicted in Fig. 2 .

#### Middle Ear Dysfunction among Mucormycosis Patients

Further analysis of mucormycosis patients revealed noteworthy findings regarding middle ear dysfunction. Among the cases studied, 14 patients experienced middle ear involvement on both sides, irrespective of the side affected by the infection. Additionally, 8 patients exhibited dysfunction on the same side as the infection (ipsilateral), while 3 experienced it on the opposite side (contralateral). However, involvement of the middle ear was absent in 5 cases ( Table 4 ).

**Table 4:** Middle ear dysfunction among mucormycosis patients ( *n* = 30)

Middle ear dysfunction	Number of patients
Bilateral	14 (46.67%)
Ipsilateral	8 (26.67%)
Contralateral	3 (10%)
No involvement	5 (16.66%)

### Acoustic Reflex Analysis: Control versus Mucormycosis

The acoustic reflex test, gauging middle ear muscle contractions to loud sounds, unveiled notable distinctions between the control and test groups. In the control group, all 60 ears displayed the reflex, signifying normal auditory function. Conversely, in the test group, only 30 out of 60 ears exhibited the reflex, with the rest showing its absence, suggesting impaired auditory function, likely attributed to mucormycosis infection ( Table 5 ).

**Table 5:** Acoustic reflex findings for both ears: control group versus test group

Acoustic reflex	Control croup	Test group (mucormycosis patients)
	Numbers of ears	Numbers of ears
Present	60	30
Absent	0	30

## Discussion

During the COVID-19 pandemic, mucormycosis became a substantial concern due to its diverse manifestations on the sinuses, orbit, lungs, and cranial cavity. 23 However, there is a lack of understanding regarding its implications on auditory health. While some case reports and series studies have briefly mentioned this aspect, comprehensive data are still scarce. 14 17 18 19 Aiming to address this knowledge deficit, the present study undertook a meticulous case-control investigation to provide insight into the often-overlooked impact of mucormycosis on the auditory system.

Audiometric analysis in the current study revealed notable differences in hearing thresholds between mucormycosis patients and healthy controls across various frequencies. These findings are consistent with previous research, 14 17 19 24 indicating a consistent pattern of decreased hearing sensitivity in individuals with mucormycosis. Biniyam et al. 14 observed moderate hearing loss in their case study, primarily attributed to chronic suppurative otitis media and associated tympanic membrane perforation, with mucormycosis potentially exacerbating the condition. Similarly, Hazarika et al. 17 observed moderate hearing loss in their case report involving middle ear mucormycosis. Another study by Jati et al. 19 reported moderately severe hearing loss and Eustachian tube dysfunction following COVID-19-associated mucormycosis despite the absence of prior otologic symptoms. Furthermore, Shah et al. 24 presented a case of mucormycosis causing facial palsy and involving atypical sites like the temporal bone, resulting in sloping hearing loss. Our study confirmed higher median thresholds, especially at higher frequencies, such as 4 and 8KHz, aligning with Jati et al. and Shah et al. 19 24 This underscores the diverse impact of mucormycosis on auditory health.

The present study also observed a significant prevalence of hearing loss among mucormycosis patients, with approximately half exhibiting some degree of loss. While previous reports primarily noted moderate to moderately severe hearing loss, 14 17 19 our study also identified mild cases. This variability could be due to factors like fungal invasion extent, individual immune responses, age, timing of diagnosis, fungal strain variations, and comorbidities.

Our study uncovered a unique trend in hearing loss among mucormycosis patients. We found that patients with left-sided mucormycosis typically exhibited left-ear hearing loss, whereas right-sided mucormycosis was associated with bilateral hearing loss. This specific pattern has not been documented in previous studies and may be attributed to the close anatomical proximity of the fungal infection to the auditory structures. Delving deeper into this phenomenon through imaging studies and histopathological analyses could provide valuable insights into the underlying mechanisms.

After investigating differences between test and control groups, efforts to identify determinants of poor auditory outcomes in mucormycosis patients yielded no statistically significant associations between hearing loss and the studied variables. However, certain trends related to mucormycosis side and type, as well as patient age suggest potential risk factors. While these trends may suggest potential risk factors for hearing impairment in mucormycosis patients, larger-scale studies are imperative to validate these observations and elucidate the underlying mechanisms.

The investigation into middle ear function via immittance analysis revealed a notably higher incidence of middle ear abnormalities in the mucormycosis group compared to the controls. While previous studies 13 14 17 18 19 documented middle ear involvement in mucormycosis, they often considered it to be a rare occurrence. In contrast, our study identified frequent middle ear involvement among mucormycosis patients, indicating a higher prevalence than previously reported. Subsequent analysis within this group of patients showed that middle ear dysfunction was predominantly bilateral, followed by ipsilateral, with less frequent contralateral involvement to the side of infection. These findings differ from earlier observations by Kumar et al., 19 who reported a predominance of ipsilateral dysfunction. The likely reason for this discrepancy was that initial investigations often missed ear involvement due to the absence of otologic symptoms and a lack of awareness among professionals that mucormycosis can affect the middle ear even without prior clinical signs. The proximity and direct accessibility of the middle ear via the Eustachian tube from the sinuses might have contributed to the observed complications. 14 17 18 19 Furthermore, half of the mucormycosis patients in this study demonstrated an absence of acoustic reflexes, indicating middle ear issues consistent with findings from previous studies. 18 19

The hearing loss and abnormal tympanograms observed among mucormycosis patients in our study suggest a potential pathophysiological link between this condition and the auditory system. Mucormycosis, renowned for its aggressive tissue invasion and rapid destruction, 1 2 3 likely exacerbates auditory complications through diverse pathways. Its infiltration of nasal and sinus mucosa can progress to the middle ear via the Eustachian tube, causing inflammation, tissue necrosis, and middle ear dysfunction. 14 18 19 In our study, middle ear dysfunction manifested as hearing loss and abnormal tympanograms without preceding otologic symptoms. Moreover, the angioinvasive trait of mucormycosis may induce thrombosis in blood vessels supplying auditory structures, causing ischemia and subsequent tissue damage. 25 26 27 This vascular compromise may extend to auditory structures, affecting the cochlea and auditory nerve, ultimately resulting in different types of hearing loss.

Overall, the present study emphasizes the critical need for increased awareness and vigilance concerning the auditory effects of mucormycosis, especially among immunocompromised individuals. Our findings indicated this condition significantly jeopardized auditory health, potentially causing hearing loss and middle ear dysfunction. Understanding the pathophysiological mechanisms underlying these auditory manifestations is crucial for early detection, intervention, and management. Further research is warranted to elucidate potential targets for therapeutic intervention and improve clinical outcomes for those affected.

Despite the comprehensive approach taken in the present study, several limitations should be acknowledged. First, the sample, though meticulously selected, was relatively small, limiting the generalizability of the findings. Second, the study, although robust, was limited to a case-control design, which inherently restricts the ability to establish causality. Additionally, the study primarily focused on a specific age group (20–49 years), potentially limiting the extrapolation of findings to other age groups. Finally, while the study identified associations between mucormycosis and auditory dysfunction, the underlying mechanisms remain unclear. Nonetheless, this study provided valuable insights into the auditory implications of this infection, laying the groundwork for future research in this field.

## Conclusion

This groundbreaking study highlighted the often-overlooked auditory implications of mucormycosis, emphasizing its significant impact on auditory health. Through a meticulous case-control analysis, we observed a notable prevalence of hearing loss and middle ear dysfunction among this population. Despite our limitations, the study underscored the importance of routine audiological evaluations for mucormycosis patients and the critical need for increased awareness and vigilance regarding mucormycosis-related auditory implications. The results of this study also suggested potential risk factors for hearing impairment, warranting further large-scale research to validate these observations and elucidate the underlying mechanisms. Overall, this research provides valuable insights into the auditory consequences of mucormycosis, paving the way for improved diagnosis, treatment, and preventive strategies to enhance clinical outcomes for affected individuals.
